# Diagnostic value of pleural fluid SMRP, CA125, MMP-7, and MMP-9 in malignant pleural effusion

**DOI:** 10.1097/MD.0000000000032759

**Published:** 2023-01-27

**Authors:** Gaohua Han, Jun Li, Xinbo Liu, Ruyue Guo

**Affiliations:** a Department of Oncology, Taizhou People’s Hospital of Nanjing Medical University, Taizhou, Jiangsu, China; b Department of Oncology, Dalian Medical University, Dalian, Liaoning, China.

**Keywords:** diagnostic, MMP-7, MMP-9, pleural effusion, SMRP

## Abstract

This study aimed to investigate the clinical value of mesothelin soluble related peptide (SMRP), cancer antigen 125 (CA125), matrix metalloproteinase-7 (MMP-7), and matrix metalloproteinase-9 (MMP-9) in benign and malignant pleural exudative effusion. A total of 105 adult patients with pleural exudative effusion admitted in our hospital from December 2019 to December 2020 were selected. Patients were divided into the benign group (n = 60) and the malignant group (n = 45) according to their condition. The levels of SMRP, CA125, MMP-7, and MMP-9 in the pleural effusion were determined by enzyme linked immunosorbent assay. Receiver operating characteristic curves were used to analyze the individual and combined predictive value of SMRP, MMP-7, MMP-9, and CA125 levels. In the malignant group, the SMRP, CA125, MMP-7, and MMP-9 levels were all significantly higher than those in benign group (*P* = .01). The detection efficiency of the 4 indicators in the combined diagnosis were higher than that of single index and combination of any 2 indices. There was a moderate positive correlation between SMRP and CA125 and MMP-7 in malignant pleural effusion. The correlation between MMP-7 and MMP-9 was moderately positive. The diagnostic efficacy of SMRP combined with CA125, MMP-7, and MMP-9 in pleural effusion for malignant pleural effusion and BPE are better than single index, which has certain clinical values for the selection of early intervention scheme for BPE patients.

## 1. Introduction

Malignant pleural effusion (MPE) is usually caused by the primary pleural malignant tumor or pleural metastatic tumor,^[1]^ which is one of the common complications in patients with advanced cancer. Many methods can be used to diagnose MPE. Pleural effusion cytological analysis is always the clinician’s first choice, but its diagnostic efficiency is far from ideal. Therefore, finding a more sensitive biomarker to diagnose MPE is of great clinical significance for early identification of malignant pleural effusion for timely and effective anti-tumor treatment. Mesothelin, as a cell-surface glycoprotein, is expressed in normal human tissues is essentially limited to the mesothelial cells lining the pleura, peritoneum, and pericardium, but highly expressed in many common cancer.^[2,3]^ Soluble mesothelin-related peptide (SMRP), is formed by mesothelin shedding from the cell surface and enter the thoracic and abdominal cavity, which has the potential value to become a biomarker for tumor diagnosis and prognosis monitoring.^[4]^ Many studies have proved that mesothelin and cancer antigen 125 (CA125) have high affinity and binding specificity, and the combination of them can promote the implantation and metastasis of cancer cells in serosal cavity.^[3,5,6]^ Matrix metalloproteinases is a multifunctional enzyme which plays a key role in process of extracellular matrix formation and degradation, and is closely related to the proliferation, invasion and migration of tumor cells. Chen et al found that Mesothelin binding to CA125 promotes cancer cell motility and invasion via matrix metalloproteinase-7 (MMP-7) Activation and mesothelin could enhance the expression of matrix metalloproteinase-9 (MMP-9) to enhance tumor invasion.^[7,8]^ As in previous related studies, CA125, SMRP, MMP-7, and MMP-9 may be correlated to some extent. The aim of this study is to investigate the diagnostic efficacy of SMRP, CA125, MMP-7, and MMP-9 in benign and malignant pleural effusion.

## 2. Materials and methods

### 2.1. General information

From December 2019 to December 2020, 105 patients with pleural effusion treated in our hospital were selected and divided into the malignant group (60 patients with malignant pleural effusion, including 43 lung cancer, 8 esophageal cancer, 3 gastric cancer, 2 ovarian cancer, 3 cancer, and 1 malignant pleural mesothelioma) and the malignant group (45 patients of benign exudative pleural effusion, including 33 pneumonia and 12 tuberculous pleurisy). Basic information of patients was collected at the time of enrollment, All of the cases and controls were enrolled consecutively.

The inclusion criteria of benign group were as follows: The patient was previously diagnosed with malignant tumor by pathology, and tumor cells or highly heterotypic cells were found in pleural effusion; Newly diagnosed patients were diagnosed as pleural primary or metastatic malignant tumors with pleural effusion by imaging diagnosis or pleural biopsy; Without local intrathoracic perfusion treatment before enrollment. The inclusion criteria of benign group were as follows: the pleural effusion of patients could be identified as exudative effusion according to the light’s criteria and malignant lesions were excluded, without any local intervention treatment before enrollment. Patients who met the above criteria were eligible for inclusion in the study. This study has passed the ethical review of the Ethics Committee of Taizhou People’s Hospital (Ethics No.: KY 201916801) and informed consent was obtained from all participants.

### 2.2. Detection method

A total of 30 mL fresh pleural effusion was collected as samples for examination at the time of the patient’s first thoracentesis and centrifuged for 20 minutes at a speed of 2500 rev/minute. The supernatant was stored in the refrigerator at −80°C in the central laboratory of our hospital for examination. The levels of SMRP, CA125, MMP-7, and MMP-9 in pleural effusion were detected by enzyme-linked immunosorbent assay were conducted in accordance with the operating specifications and kit instructions. These reagents were purchased from Santa Cruz Biotechnology.

### 2.3. Statistical analysis

SPSS 26.0 statistical software (SPSS Inc., Chicago, IL) was used to processed the data. For the descriptive analysis, the frequencies of qualitative variables were used, as well as mean ± standard deviation (SD) expressed for normally distributed quantitative variables and medians (25th and 75th quartiles) expressed for non-Gaussian quantitative variables. Groups were compared using the student *t* test for normally distributed variables and using the non-parametric Mann–Whitney *U* test for non-Gaussian variables. The correlation coefficient between variables in malignant pleural effusion group was performed using Pearson correlation test. Receiver operating characteristic (ROC) curves and the areas under the curves (AUCs) were used to evaluate these indexes diagnostic value. Sensitivity, specificity and Youden index were also analyzed. The optimal cutoff points were established based on their maximum Youden index. When using more than 1 variable calculated of the ROC curve Logistic regression analysis was used to calculate the prediction probability, and then ROC curve was drawn based on the prediction probability. All *P* values were 2-sided. A statistically significant difference was set when *P* values <.05.

## 3. Results

### 3.1. Characteristics of patients

A total of 105 patients were enrolled in our study. There were 60 patients in malignant group including 32 males and 28 females, aged from 44 to 86 years old, with the average age of (65.64 ± 9.64) years old. There were 45 patients in benign group including 28 males and 17 females, aged from 17 to 92 years old, with the average age of (68.97 ± 17.17) years old. There were no significant differences in gender and age between the 2 (*P* = .01) (Table 1).

**Table 1:** Baseline characteristic of malignant and benign group.

Variable	Malignant group (N = 60)	Benign group (N = 45)	*t*/*χ*^2^	*P* value
Age (yr)	66.17 ± 9.84	67.37 ± 17.61	−0.44	.66
Gender				
Male	32	28	0.384	.53
Female	28	17		

### 3.2. Comparison of SMRP, CA-125, MMP-7, and MMP-9 levels in benign and malignant pleural effusion

We analyzed the experimental data between benign and malignant pleural effusion group. The expression level of CA125 in malignant pleural effusion group was 142.64 (101.15, 178.72) U/mL, and that in benign pleural effusion group was 92.64 (81.28, 106.08) U/mL, The expression level of MMP-7 in malignant pleural effusion group was (15.83 ± 5.34) ng/mL, and the level of MMP-7 in benign pleural effusion group was (10.82 ± 2.63) ng/mL, The expression level of SMRP in malignant pleural effusion group was 0.82 (0.63, 1.24) pmol/mL, and that in benign pleural effusion group was 0.65 (0.56, 0.93) pmol/mL. The expression level of MMP-9 in malignant pleural effusion group was (109.28 ± 27.39) ng/mL, and that in benign pleural effusion group was (72.66 ± 16.56) ng/mL. We found that in the malignant group, the levels of SMRP, CA125, MMP-7, and MMP-9 in pleural effusion were higher than those in the benign group, and all of which had a statistical significance (*P* = .01) (Table 2).

**Table 2:** Expression of SMRP, CA-125, MMP-7, and MMP-9 in pleural effusion.

	Malignant group	Benign group	*Z*/*t*	*P* value
SMRP (pmol/mL)	0.82 (0.63, 1.24)	0.65 (0.56, 0.93)	−2.3	.01
CA125 (U/mL)	142.64 (101.15, 178.72)	92.64 (81.28, 106.08)	−5.6	.01
MMP-7 (ng/mL)	15.83 ± 5.34	10.82 ± 2.63	5.9	.01
MMP-9 (ng/mL)	109.28 ± 27.39	72.66 ± 16.56	7.9	.01

CA125 = cancer antigen 125, MMP-7 = matrix metalloproteinase-7, MMP-9 = matrix metalloproteinase-9, SMRP = mesothelin soluble related peptide.

### 3.3. Comparison of differential diagnostic value of SMRP, CA125, MMP-7, and MMP-9

**Figure 1.**
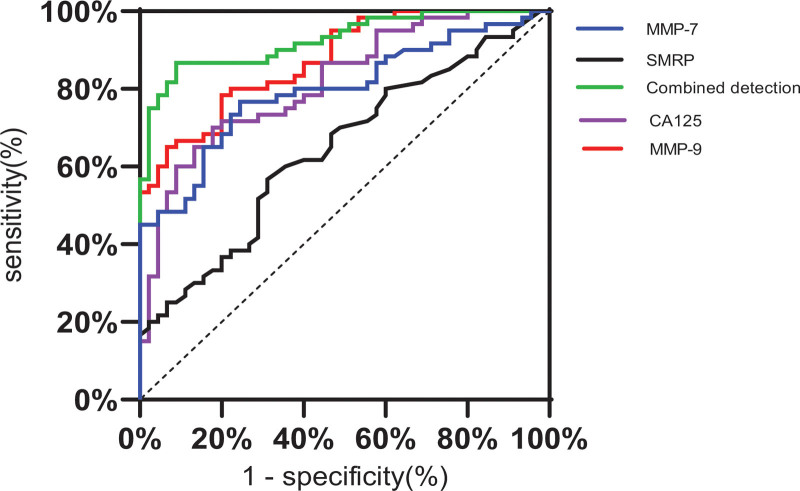
ROC curves of SMRP, CA125, MMP-7, MMP-9, and combined detection. The area under the ROC curve (AUC) of SMRP was 0.644, that of CA125 was 0.816, that of MMP-7 was 0.796, that of MMP-9 was 0.874, and that of combination of the 4 indicators was 0.926. AUC = area under the curve, CA125 = cancer antigen 125, MMP-7 = matrix metalloproteinase-7, MMP-9 = matrix metalloproteinase-9, ROC = receiver operating characteristic, SMRP = mesothelin soluble related peptide.

**Figure 2.**
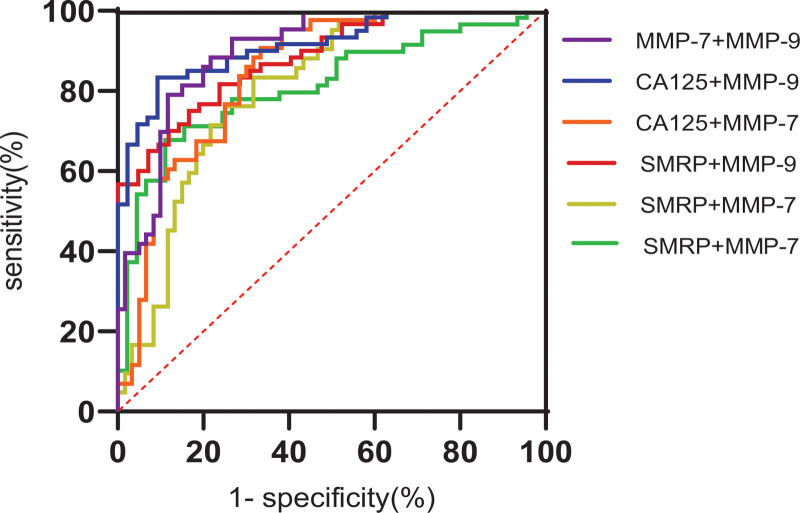
ROC curves of the combination of any two indices. The area under the ROC curve (AUC) of SMRP + CA125 was 0.814, that of SMRP + MMP-7 was 0.813, that of SMRP + MMP-9 was 0.8885, that of CA125 + MMP-7 was 0.849, that of CA125 + MMP-9 was 0.914, and that of MMP-7 + MMP-9 was 0.901. AUC = area under the curve, CA125 = cancer antigen 125, MMP-7 = matrix metalloproteinase-7, MMP-9 = matrix metalloproteinase-9, ROC = receiver operating characteristic, SMRP = mesothelin soluble related peptide.

The ROC curves of SMRP, CA125, MMP-7, and MMP-9 alone or in combination were drawn. According to the area under the curve (AUC) results, the combined detection effect of the 4 indicators was better than that of a single indicator (Figs. 1 and 2). The sensitivity and specificity of the combined detection of malignant pleural effusion were the highest (Table 3).

**Table 3:** Differential diagnostic value of SMRP, CA125, MMP-7, and MMP-9 in pleural fluid.

	AUC (95% CI)	Cut-off	Sensitivity (%) (95% CI)	Specificity (%) (95% CI)
SMRP	0.644 (0.54–0.75)	0.77	56.7 (44.1–86.4)	68.9 (54.3–80.5)
CA125	0.816 (0.74–0.89)	108.58	82.2 (68.7–90.7)	71.1 (57.5–80.1)
MMP-7	0.796 (0.71–0.88)	12.29	76.7 (64.6–85.6)	75.6 (61.3–85.7)
MMP-9	0.874 (0.81–0.93)	87.83	78.3 (66.4–86.9)	80 (66.2–89.1)
SMRP + CA125	0.814 (0.72–0.89)	43.9%	68.3 (53.9–0.9)	88.9 (66.4–86.9)
SMRP + MMP-7	0.813 (0.73–0.89)	48.3%	75 (62.2–83.9)	77.8 (61.3–85.8)
SMRP + MMP-9	0.885 (0.82–0.94)	38.6%	75 (62.7–84.2)	83.3 (69.4–91.7)
CA125 + MMP-7	0.849 (0.77–0.92)	36.7%	66.7 (50.2–77.6)	91.1 (70.1–89.4)
CA125 + MMP-9	0.914 (0.86–0.96)	39.2%	83 (71.9–90.7)	89 (78.4–96.3)
MMP-7 + MMP-9	0.901 (0.84–0.96)	56.7%	88.3 (75.5–94.9)	80 (66.4–86.9)
Combination of the 4	0.926 (0.87–0.97)	41.2%	86.7 (75.8–93.1)	91.1 (79.3–96.5)

AUC = area under the curve, CA125 = cancer antigen 125, MMP-7 = matrix metalloproteinase-7, MMP-9 = matrix metalloproteinase-9, SMRP = mesothelin soluble related peptide.

### 3.4. Correlation of SMRP, CA125, MMP-7, and MMP-9 in malignant pleural effusion

Pearson method was used to analyze the correlation between CA125, MMP-7, and MMP-9, and Spearman rank correlation analysis was used to analyze the correlation between SMRP and other indicators. The results showed that SMRP, CA125, and MMP-7 were moderately positively correlated in malignant pleural effusion. There was a moderate positive correlation between MMP-7 and MMP-9, and a weak correlation between MMP-9 and SMRP and CA125 (Table 4).

**Table 4:** Correlation of SMRP, CA125, MMP-7 and MMP-9 in malignant pleural effusion.

	CA125	MMP-7	MMP-9
SMRP	0.42 (*P* < .01)	0.41 (*P* < .01)	0.34 (*P* < .01)
CA125	–	0.51 (*P* < .01)	0.19 (*P* = .14)
MMP-7	–	–	0.45 (*P* < .01)

CA125 = cancer antigen 125, MMP-7 = matrix metalloproteinase-7, MMP-9 = matrix metalloproteinase-9, SMRP = mesothelin soluble related peptide.

## 4. Discussion

Malignant pleural effusion can occur in 15% cancer patients during the course of disease. With the increasing incidence of cancer worldwide and the improvement of overall survival rate, the incidence of malignant pleural effusion may increase year by year. Early identification of MPE is the premise of making a correct and appropriate diagnosis and treatment plan and evaluating prognosis.^[9,10]^

Current studies have shown that mesothelin can play a role in the process of tumor cell proliferation, tumor invasion and metastasis, and tumor drug resistance. The isomer SMRP generated after its hydrolysis can be dissolved in pleural and peritoneal fluid and blood and detected. These characteristics make SMRP promising to be a tumor diagnostic biomarker. SMRP has been widely studied as biomarkers for the diagnosis and prognostic monitoring of malignant mesothelioma and ovarian cancer.^[11,12]^ Fran et al^[13]^ showed that the high expression of SMRP in pleural fluid is of guiding significance for the diagnosis of malignant pleural mesothelioma with negative cytology. The results of a meta-analysis showed that the presence of SMRP in pleural effusion could support the diagnosis of mesothelioma.^[14]^ However, there are few reports on the differentiation of benign and malignant pleural effusion by SMRP. Fu Wang et al^[15]^ showed that the expression of SMRP in MPE was higher than that in benign pleural effusion, but the results were not statistically significant. The results of our study showed that SMRP in the malignant group was significantly higher than that in the benign group (*P* = .01), with a specificity of 56.7% and a sensitivity of 68.9%. Compared with the widely used tumor biomarkers, SMRP alone could not improve the diagnostic ability of distinguishing malignant pleural effusion, and the combined detection with other biomarkers could be considered.

CA125 is a common tumor biomarker, which is present in normal tissues, such as peritoneal/pleural mesothelial tissue cell surface, reproductive organ epithelium, etc.^[16]^ CA125 can be increased in patients with gynecological tumors, lung cancer and other malignant tumors, and it is related to clinical stage and prognosis.^[17–19]^ When the serous cavity is stimulated by inflammation or malignant transformation, CA125 can be elevated in serous cavity effusion. Elevated CA125 can assist in the differential diagnosis of malignant pleural effusion and tuberculous pleural effusion, but its specificity in the diagnosis is low.^[20,21]^ Our study showed that the expression of CA125 in MPE was significantly higher than that in BPE. The AUC of CA125 was 0.816, the specificity of diagnosis was 71.1%, and the sensitivity was 82.2%, indicating that its sensitivity was high and specificity was low. This is consistent with the conclusion of a meta-analysis that CA125 has some value in differentiating benign and malignant pleural effusion and is a tumor marker with high sensitivity but low specificity.^[22]^ Related studies have shown that mesothelin can promote the survival of suspended tumor cells in ascites and specifically bind to CA125 with high affinity, mediate the adhesion of cancer cells to the mesothelium, increase the ability of tumor cells to invasion the mesothelium, and thus promote the extensive metastasis of cancer cells in the serous cavity.^[3,5]^ In this study, the correlation analysis of SMRP and CA125 in malignant pleural effusion showed that there was a moderate positive correlation between them, suggesting that the expression level of mesothelin may have a certain correlation with the malignant degree of pleural effusion, and the 2 may play a synergistic role in the process of pleural cavity metastasis and pleural effusion formation, and further mechanism research is needed. At present, there are monoclonal antibodies (such as MORAb-009) targeting mesothelin in clinical trials, which can inhibit the binding of mesothelin to CA125.^[23]^ We might hazard a guess that this could lead to new approaches to the treatment of MPE.

MMPs play an important role in the process of tumor metastasis by degrading extracellular matrix to destroy the histological barrier around tumors and promote the invasion of cancer cells into adjacent tissues.^[24]^ The results of this study showed that the levels of MMP-7 and MMP-9 in malignant pleural effusion were significantly higher than those in benign pleural effusion. The AUC of MMP-7 was 0.796, the specificity of diagnosis was 75.6%, and the sensitivity was 76.7%. The AUC of MMP-9 was 0.874, the specificity of diagnosis was 78.9%, and the sensitivity was 77.8%. The diagnostic specificity and sensitivity of the 2 were basically the same, and the sensitivity of MMP-9 was slightly higher than that of MMP-7, which may have higher diagnostic value, which is consistent with the results of previous studies.^[25–27]^ This study selected the benign pleural effusion group were caused by pneumonia or tuberculosis exudative pleural effusion, and the result accords with the research in a previous study, but as a result of the test cases is less, not separate pneumonia and tuberculosis group comparison, such as increasing sample size further refine grouping, might be more accurate understanding of MMP-7, MMP-9 in the diagnosis of benign and malignant breast water value.

In addition, this study showed that the sensitivity (91.1%), specificity (86.7%) and area under the ROC curve of the combined detection of SMRP, CA125, MMP-7, and MMP-9 were higher than those of single detection, suggesting that the combined detection has better diagnostic efficacy in the differentiation of benign and malignant pleural effusion. The combination of MMP-9 with CA125 and MMP-7 also has high sensitivity and specificity. Considering the clinical cost and the popularity of CA125 detection, MMP-9 combined with CA125 may have higher clinical application value. The correlation analysis of SMRP, CA125, and MMP-7/9 in malignant pleural effusion showed that there was a moderate positive correlation between SMRP, MMP-7, and CA125. Combined with previous studies,^[7,27]^ CA125 was released from the cell surface through MMP-7 and MMP-9. Tumor cells co-expressing mesothelial and CA125 can increase the synthesis of MMP-7 and promote tumor metastasis. We consider that SMRP, MMP-7, and CA125 may play a mutually promoting and synergistic role in the process of pleural metastasis and pleural effusion formation, but further mechanism research is needed.

This study only preliminaries that SMRP, CA125, and MMP-7/9 are different between benign and malignant pleural effusion, which may have certain significance for the differentiation of benign and malignant pleural effusion. However, sample size was relatively small in this study, and the comparison of malignant and benign pleural effusion is not further refined and classified, which may have a certain impact on the final results. Further studies with large samples are needed to find a more suitable clinical application range.

## Author contributions

**Conceptualization:** Gaohua Han, Jun Li.

**Data curation:** Jun Li.

**Formal analysis:** Xinbo Liu.

**Methodology:** Gaohua Han, Jun Li.

**Resources:** Gaohua Han.

**Software:** Jun Li, Xinbo Liu, Ruyue Guo.

**Supervision:** Gaohua Han.

**Validation:** Jun Li.

**Visualization:** Ruyue Guo.

**Writing – original draft:** Jun Li.

**Writing – review & editing:** Jun Li, Gaohua Han.
